# Impact of Outborn/Inborn Birth Status of Infants Born at <29 Weeks of Gestation on Neurodevelopmental Impairment: A Nationwide Cohort Study in Korea

**DOI:** 10.3390/ijerph191811718

**Published:** 2022-09-16

**Authors:** In Young Cho, Hye Mi Lee, Sae Yun Kim, Eun Sun Kim

**Affiliations:** 1 Department of Pediatrics, Yeouido St. Mary’s Hospital, College of Medicine, The Catholic University of Korea, Seoul 07345, Korea; 2 Department of Pediatrics, Kangwon National University School of Medicine, Chuncheon 24341, Korea

**Keywords:** inborn, outborn, perinatal retrieval system, preterm

## Abstract

This study designed to evaluate the short- and long-term outcomes of outborn and inborn preterm infants enhancing the regional perinatal system in South Korea. It is a prospective cohort study of the Korean neonatal network database for infants born at <29 weeks of gestation between 2013 and 2015. Of 2995 eligible infants, 312 were outborn, and 976 completed the assessment of long-term outcome at 18–24 months of corrected age. The mean gestational age was significantly younger in outborn infants than in inborn infants (*p* = 0.004). The mean Apgar score at 5 min was higher in inborn infants (*p* = 0.046). More inborn preterm infants died before discharge (*p* < 0.001); however, most of the other short-term outcomes occurred significantly more often in outborn infants than in inborn infants. The outborn infants had higher odds of neurodevelopmental impairment (adjusted odds ratio (aOR) 2.412, 95% confidence interval (CI) 1.585–3.670), cerebral palsy (aOR 4.460, 95% CI 2.249–8.845) and developmental impairment (aOR 2.238, 95% CI 1.469–3.408). In preterm infants, the location of birth may be a key factor influencing short- and long-term outcomes. Thus, to provide adequate care and efficiently allocate medical resources to high-risk preterm infants, nationwide regional perinatal systems need to be improved and standardized.

## 1. Introduction

With improvements in neonatal intensive care unit (NICU) practices, the survival rate of preterm infants has increased significantly, as well as the short- and long-term outcomes of extremely preterm (EPT) infants [1,2]. EPT infants are at the maximal risk of mortality and morbidity; therefore, they require highly specialized multidisciplinary medical support: subspecialized neonatologists, experienced nursing staffs, pediatric surgeons, monitoring equipment for vital signs, and laboratory facilities for microsample. To address these needs, providing optimal support at immediate birth is key to improve the short- and long-term outcomes of EPT infants. Therefore, access to initial neonatal care is very important.

Like many other countries, in South Korea, expectant mothers with a high risk of premature delivery are preemptively transferred to tertiary hospitals where neonatal intensive care is available for high-risk infants. However, due to the nature of premature birth, transferring high-risk mothers to tertiary centers is not always possible, and infants are often born in a small birth center of regional obstetric clinics. Infants, especially EPT infants born outside tertiary hospitals, have a higher risk of mortality and morbidity, including long-term NDIs, than inborn infants [3,4,5,6,7,8].

The American Academy of Pediatrics and the American College of Obstetrics and Gynecologists Guidelines for Perinatal Care recommend that maternity hospitals be located to provide adequate care for high-risk pregnancies and infants in specific places [9,10]. In Western countries and Japan, the regionalization of perinatal care is already settled. Maternal and neonatal care is stratified into three levels of complexity; high-risk pregnant women are referred to centers with appropriate personnel and resources according to the degree of risk and severity of illness [9]. Transporting women before delivery to tertiary centers has substantially improved neonatal outcomes including mortality [11]. Therefore, expectant mothers with high-risk pregnancies should be transferred to tertiary hospitals with NICUs preemptively. However, because the labor progressed rapidly and the high-risk pregnant women could not find any tertiary hospitals with capacity, therefore, several neonates are transferred to tertiary referral perinatal centers for proper management, after birth. Although there has been a consensus about the need for a regionalized perinatal care system, implementation of standardized medical services has not been provided for each level of care, and the definition of each level has not been properly established in South Korea. Thus, solving perinatal problems remains a crucial issue for maternal and neonatal health.

However, literature comparing neonatal and neurodevelopmental outcomes of outborn infants is relatively lacking. A cohort study from Japan disclosed that short-term neonatal morbidities and NDI at 3 years of age were significantly higher in outborn infants than in inborn infants [12]. Amer et al. reported that the mortality rate and NDI were significantly higher in outborn infants than in inborn infants admitted to the Canadian NICU [5]. Meanwhile, Bolbocean et al. reported that non-ambulatory cerebral palsy (CP) was not associated with where the neonates were born (outborn or inborn) in a cohort of 360 infants from Canada [13]. In addition, in an Australian cohort study, there was no significant difference in NDI at 2–3 years of age between outborn and inborn EPT infants born between 1998 and 2004 [14]. The objective of our study was to evaluate whether the place of birth (in hospital or out of hospital) of EPT infants influences short- and long-term outcomes based on the Korean Neonatal Network (KNN) database.

## 2. Materials and Methods

### 2.1. Study Design and Participants

The KNN, entrenched by the Korean Society of Neonatology and the Korea Centers for Disease Control and Prevention in 2013, is a nationwide prospective cohort registry of very-low-birth-weight (VLBW) infants admitted to the 75 participating NICUs, covering >90% of VLBW infants in Korea [15]. Each participating hospital’s institutional review board (IRB) approved data collection for the KNN. The KNN data management committee regularly monitored all data, and all methods were performed under relevant guidelines and regulations.

**Figure 1:**
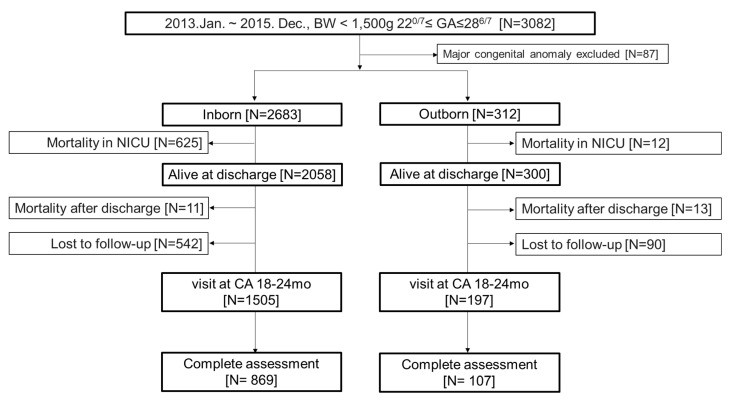
Flow chart of inborn and outborn infants from birth to follow-up. Analyzed data (*n* = 2995: inborn, 2683; outborn, 312) were obtained from the Korean Neonatal Network Database (2013–2015). GA, gestational age; CA, corrected age.

Among the registered population, infants with gestational age (GA) between 22^0/7^ and 28^6/7^ weeks, and born in participating hospitals from 1 January 2013 to 31 December 2015, were eligible for inclusion. EPT infants born and admitted to the same participating hospital immediately served as the inborn group, and EPT infants transferred to different hospitals after birth served as the outborn group. Moreover, those who survived until NICU discharge and were followed up until 18–24 months of corrected age at affiliated outpatient clinics were selected from the KNN registry for long-term outcome evaluation (Figure 1).

The short-term assessment included a maternal history and a physical examination for an infant. For the long-term assessment, the follow-up protocol for evaluating growth and development consisted of physical evaluations and developmental assessments at 18–24 months of corrected age for surviving infants at each participating hospital. Infants who visited the follow-up clinic assessed motor development, eye and ear status, and underwent comprehensive developmental evaluation using the Bayley Scales of Infant Development, Second Edition (BSID-II), Bayley Scales of Infant and Toddler Development, Third Edition (BSID-III) [16], and/or the Korean Developmental Screening Test (K-DST) [17].

### 2.2. Definitions

The GA was calculated based on the last menstrual period. Maternal hypertension includes gestational hypertension, pregnancy-induced hypertension, and chronic hypertension. Maternal diabetes was defined as the diagnosis of gestational diabetes or overt diabetes during pregnancy. Histological chorioamnionitis was defined as described by Yoon et al. [18]. Premature rupture of membranes was defined as rupture of membranes more than 24 h before delivery. More than one dose of antenatal corticosteroid (ACS) administered to the mother at any time before delivery was considered as ACS administration. Small for gestational age (SGA) was named as a birth weight under the 10th percentile for GA and sex [19]. Respiratory distress syndrome (RDS) was determined as a radiologic and clinical diagnosis with surfactant replacement therapy. The National Institute of Child Health Workshop criteria for bronchopulmonary dysplasia (BPD) were used [20]. Patent ductus arteriosus (PDA) was confirmed by echocardiographic and clinical findings. Sepsis was determined based on positive blood culture results. Intraventricular hemorrhage (IVH) was designated as a Papile classification of grade 3 or 4 on cranial ultrasonography [21]. Periventricular leukomalacia (PVL) was described as cystic or non-cystic periventricular white matter injury on cranial imaging observed at any time. Severe brain injuries include IVH III or IV, and PVL. Necrotizing enterocolitis (NEC) was defined according to Bell’s classification as stage II or higher [22]. Severe retinopathy of prematurity (ROP) was denoted as stage 3 or higher, or retinopathy requiring treatment with laser or antivascular endothelial growth factor [23,24]. NICU mortality is determined as the death of an infant before NICU discharge.

A diagnosis of CP was made using standard definitions [25], and the degree of functional impairment was determined by the Gross Motor Function Classification System (GMFCS) [26]. Visual impairment was defined as bilateral blindness as diagnosed by an ophthalmologist. From hearing assessment results and history, hearing impairment was designated as hearing loss or the need for hearing aids or cochlear implants. Developmental impairment was defined when at least one of the following criteria was met: (1) BSID-II Mental Development Index or BSID-II Psychomotor Development Index score < 70; (2) BSID-III cognitive composite score, language composite score, motor composite score, or general adaptive composite score < 70; and (3) K-DST score below 2 standard deviations. NDI was defined as at least one of the following: CP with GMFCS ≥ III, developmental impairment, bilateral hearing impairment, or bilateral visual impairment.

### 2.3. Statistical Analysis

Maternal characteristics, infant characteristics, short- and long-term outcomes were compared between the outborn and inborn groups using the Pearson chi-square test or Fisher exact test for categorical variables and the Student’s t-test or Wilcoxon rank test for parametric and nonparametric continuous variables, as appropriate. Univariate and multivariate logistic analyses were performed for primary and secondary outcomes. For multivariable analysis, GA and 5 min Apgar scores were adjusted to calculate aORs and 95% CIs. Statistical analysis was conducted using SAS version 9.4 (SAS Institute, Cary, NC, USA) and SPSS version 25 (IBM Corp, Armonk, NY, USA) with a significance level of 0.05.

## 3. Results

The population comprised 2995 infants (312 (10.4%) outborn and 2683 (89.6%) inborn) from a total of 3082 admissions during 2013–2015. Before discharge, 637 neonates died: 625 and 12 neonates in the inborn and outborn groups, respectively. Twenty-four children died before the follow-up, 632 were lost to follow-up, 1702 visited or were connected by phone, and ultimately 976 children completed the follow-up assessment (Figure 1). There were few differences in perinatal and neonatal characteristics between the outborn and inborn groups. The GA at birth of outborn infants was lower than that of inborn infants (26^1/7^ ± 1^4/7^ vs. 26^4/7^ ± 1^5/7^ *p* = 0.004). Outborn infants had lower 5 min Apgar scores than inborn infants (5.9 ± 1.9, 6.1 ± 1.9, *p* = 0.046) (Table 1).

Outborn infants had higher rates of sepsis, PDA requiring surgical treatment, severe brain injury or IVH 3 and 4, NEC, BPD, and severe ROP. The outborn group stayed longer in the NICU than the inborn group; the mean length of stay was 106.8 ± 56.6 days in the outborn group and 75.7 ± 46.1 days in the inborn group (*p* < 0.001). Of note, 23.3% (625/2683) of inborn infants and 3.8% (12/312) of outborn infants died before discharge (*p* < 0.001). Multivariate logistic regression analysis performed after adjusting for confounders (GA and 5 min Apgar score) showed that outborn infants had higher odds of sepsis (adjusted OR (aOR), 1.891; 95% confidence interval (CI) 1.482–2.412; *p* < 0.001), PDA requiring surgical treatment (aOR, 2.436; 95% CI, 1.872–3.169; *p* < 0.001), severe brain injury (aOR, 1.510; 95% CI, 1.154–1.977; *p* = 0.003), NEC (aOR, 2.351; 95% CI, 1.706–3.239; *p* < 0.001), and severe ROP (aOR, 1.606; 95% CI, 1.199–2.151; *p* = 0.001) (Table 2).

In contrast to NICU death trends, a higher proportion of outborn infants died after discharge and before the follow-up visit compared to inborn infants: 13/222 (5.9%) in the outborn group and 11/2141 (0.5%) in the inborn group. After adjusting for confounding factors, outborn infants showed higher odds of mortality after discharge than inborn infants (aOR, 15.658; 95% CI 6.840, 35.844; *p* < 0.001). The composite outcome of NDI or death was higher in the inborn infants; however, these did not reach statistical significance. Proportions of NDI, CP, and developmental impairment were higher in outborn infants than in inborn infants. Multivariate logistic regression analysis performed after adjustment showed that outborn infants had higher odds of NDI (aOR, 2.412; 95% CI 1.585–3.670; *p* < 0.001), CP (aOR, 4.460; 95% CI, 2.249–8.845; *p* < 0.001), and developmental impairment (aOR 2.238; 95% CI 1.469–3.408; *p* < 0.001) (Table 3).

The characteristics of infants without follow-up data are shown in Table 4. Their data were more likely to be outborn and had an older GA and heavier birth weight. The analyses include a higher proportion of primiparous women among the mothers of infants. In addition, there were more women with assisted conception among the mothers of infants who were included in the analyses. Infants without follow-up data showed a higher Apgar score, body temperature, and pH at admission.

## 4. Discussion

To the best of our knowledge, this is the first large, nationwide cohort study that explored the short- and long-term outcomes of EPT infants based on location of birth in South Korea. The current study has several noticeable findings that analyzed a national population-based multicenter contemporary cohort. First, we found that the outborn group had a significantly lower mortality rate before discharge and a higher mortality rate after discharge compared with that in the inborn group. Other short-term morbidities, such as sepsis, PDA requiring surgical treatment, severe brain injury, NEC, moderate-to-severe BPD, and severe ROP, occurred more often in outborn infants than in inborn infants. Second, from the perspective of long-term outcomes, outborn infants had significantly higher odds of NDI, CP, and developmental impairment than inborn infants.

### 4.1. Short- and Long-Term Neonatal Outcomes

In our cohort, the infants of the outborn groups were significantly younger in GA and had lower 5 min Apgar scores compared to the infants of the inborn group. However due to the possibility of imprecise recollection of obstetric history and the small mean difference of GA, it could not be conclusively interpreted that the outborn infants were born at younger GA. No significant differences were observed among the remaining factors. If only the background factors were considered, no differences between the two groups would be expected. However, we found that the outborn survivors were more afflicted with most of the short-term in-hospital health outcomes, except mortality, even after adjustment. These results are in line with those of the previous studies. Traditionally, EPT infants born in NICUs of tertiary hospitals have been shown to have better health outcomes than those born outside these facilities [27].

Amer et al. reported higher rates of death or NDI and CP in outborn infants than in inborn infants [5]. Similarly, Boland et al. reported higher rates of PVL in outborn infants than in inborn infants in Victoria, Australia [28]. Our study was consistent with the previous findings as we also found higher odds of developing NDI in outborn infants. The major contributors were CP and developmental impairment. The higher incidence of short-term morbidities with better survival rate in outborn infants would also result in a higher NDI risk. Specifically, CP in outborn infants may reflect a higher incidence of severe brain injury and IVH. Furthermore, potential hemodynamic instability during transport can affect the NDI of outborn infants [29].

### 4.2. High Inborn Mortality

In the current study, the mortality after discharge up to follow-up was consistent with previous studies which have shown that the inborn EPT infants have lower mortality rates than the outborn EPT infants [5,30]. Infants with complicated or severe diseases which were not able to be treated at the hospital where they were born were transferred to a referral hospital. Therefore, outborn infants tend to have more severe conditions, resulting in higher morbidity and mortality rates than inborn infants.

**Figure A1:**
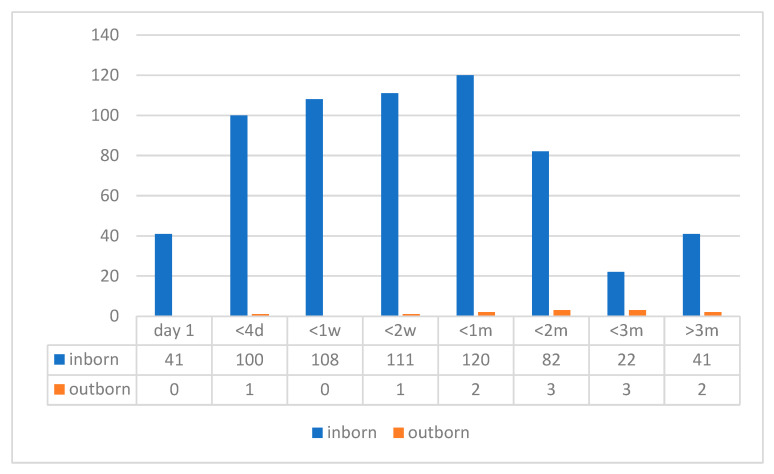
The time of death of infants who died during the study period. More than half of inborn infants died within 2 weeks after birth.

However, the in-hospital mortality during NICU admission or combined mortality of before and after discharge demonstrated the opposite outcome. The aORs were 0.073 and 0.283 in the outborn group compared with that in the inborn group, in-hospital mortality and combined mortality, respectively (Table 2 and Table 3). Usually, high-risk preterm infants can only be transferred after initial stabilization because of many dangerous factors immediately after birth. As shown in the current study, more than half of the infants died within two weeks after birth (Figure A1). Outborn infants admitted to NICUs of referral hospitals might have been a favored group, because the sickest infants could not have been transferred [29]. Furthermore, in South Korea, the regional perinatal system of neonatal transport is absent, and the transfer of high-risk infants with skilled doctors and professional equipment is rare [31]. Therefore, there were a number of high-risk infants who could not be transferred after being delivered in small hospitals.

### 4.3. Leveling of NICU

Regionalized perinatal and neonatal retrieval care has been established and systemized in the US and Japan [32,33]. However, a nationwide regionalization system for maternal or neonatal care is yet to be established in South Korea. According to data from Statistics Korea, there were 567 institutions providing delivery services nationwide: 41 tertiary general hospitals, 86 general hospitals, 145 primary obstetric hospitals, 279 maternity clinics, and 16 midwives in 2018 [31]. Indeed, in South Korea, NICUs in tertiary general hospitals provide subspecialty intensive care, equivalent to level III or IV in the United States. The NICUs in general hospitals are designed to deliver specialty care (level II in the US), and the primary obstetric hospitals or maternity clinics provide basic care for newborn nurseries (level I in the US).

Nearly half of the medical institutions are located in the Seoul metropolitan city area; among them, the top five mega-hospitals in Korea are located in Seoul. High-risk infants or women with high-risk pregnancies require highly experienced neonatal healthcare providers, specialized equipment, and the clinical setting. Therefore, some patients must be referred to a mega-hospital, because regional hospitals cannot provide proper management by specialists. Thus, women with high-risk pregnancies living outside the Seoul metropolitan city area visit a mega-hospital in Seoul to receive highly specialized prenatal care. Because of the unexpected precipitous labor, high-risk deliveries tend to occur outside the follow-up mega-hospital. Therefore, based on the condition of the neonates, high-risk infants must be transferred to other hospitals after birth. Newborn infants are generally unstable immediately after birth, and transportation of newborn infants without initial stabilization has worse consequences. Therefore, even if the initial stabilization takes time, it is desirable to transfer infants after sufficient care has been provided.

Hence, proper leveling, networking, and regionalized retrieval of neonatal and perinatal care are necessary. Unfortunately, a nationwide standard has not been established for the transportation of high-risk infants, including special ambulances with transport incubators and the presence of doctors and neonatal nursing staff in South Korea. Improvements in the nationwide regionalized perinatal retrieval system, such as optimizing transportation to tertiary centers, can improve the outcomes of high-risk infants, because several factors at birth and during transport play a role in the health risks of outborn high-risk infants.

One of the strengths of the current study was the large population-based cohort representing over 90% of South Korean NICU admissions and standardized assessments at 18–24 months of corrected age. However, this study has some limitations. First, 21.1% (632/2995) of the infants were lost to follow-up and a small portion of infants (976/1702) completed the NDI assessment at 18–24 months of corrected age. We compared the baseline characteristics between the infants included in the analysis and those without follow-up data. The proportion of outpatients was high among infants without follow-up data. There were some difficulties in accessing the long-term outcomes, because the distance and sociocultural factors may affect the differences in follow-up rates between the inborn and outborn groups. There is a higher likelihood of an adverse outcome, which means that the true risk for outborn infants could be higher than that in the current study. Second, we used three types of tests for developmental outcomes: the K-DST, BSID-II, and BSID-III. In all three, although these are very reliable tests which have credible proven criteria for developmental impairment, there was a lack of consistency in evaluation. Third, stillbirths and neonatal deaths who could not be admitted to NICUs were not included in our study. These populations may affect the results of our analyses to some extent.

## 5. Conclusions

Our study revealed a significantly higher incidence of sepsis, surgical PDA, severe brain injury, high-grade IVH, NEC, and severe ROP in outborn infants. Interestingly, the in-hospital mortality rate was higher in inborn infants, whereas the mortality rate after discharge from the NICU was higher in the outborn group. Outborn infants experienced CP and developmental impairment more frequently than inborn infants. From this study, we can conclude that location of birth may influence the short- and long-term outcomes in preterm infants. To improve the quality of the analysis of the adverse outcomes of outborn infants, we need to obtain more accurate data on outborn preterm infants. In addition, more effort is required to improve the perinatal care by standardizing nationwide perinatal retrieval systems for selecting birth hospitals and providing effective transport systems when transfer to tertiary centers is needed.

## Figures and Tables

**Table 1:** Antenatal and perinatal characteristics.

Characteristics	Inborn (*n* = 2683)	Outborn (*n* = 312)	*p* Values
**Maternal Characteristics**			
Maternal age, years	32.8 ± 4.2	33.0 ± 4.4	0.412
Maternal age > 35 y	630 (23.5%)	73 (23.4%)	0.974
Primipara	1531 (57.1%)	194 (62.2%)	0.083
Assisted conception	595 (22.2%)	71 (22.8%)	0.829
Multiple pregnancy	853 (31.8%)	103 (33.0%)	0.662
Maternal Hypertension	322 (12.0%)	29 (9.3%)	0.159
Maternal diabetes	189 (7.0%)	14 (4.5%)	0.089
Oligohydramnios	342/2424 (14.1%)	33/256 (12.9%)	0.593
Any antenatal steroid	2109/2652 (79.5%)	229/289 (79.2%)	0.909
Chorioamnionitis	992/2268 (43.7%)	111/232 (47.8%)	0.238
Premature rupture of membrane	544/2617 (20.8%)	91/289 (21.1%)	0.899
Delivered by Caesarian section	1911 (71.2%)	210 (67.3%)	0.150
**Infantile Characteristics**			
Birth weight, gram	907.1 ± 246.1	885.2 ± 212.9	0.132
Small for gestational age	215 (8.0%)	19 (6.1%)	0.231
Gestational period, week	26^4/7^ ± 1^5/7^	26^1/7^ ± 1^4/7^	0.004
Male gender	1395 (52.0%)	164 (52.6%)	0.849
AS, 1 min	3.8 ± 1.9	3.7 ± 1.9	0.390
AS, 5 min	6.1 ± 1.9	5.9 ± 1.9	0.046
AS < 7 at 5 min	1330/2668 (49.9%)	168/305 (55.1%)	0.083
BT at admission	36.0 ± 0.7	36.0 ± 0.7	0.645
pH at admission	7.26 ± 0.12	7.25 ± 0.14	0.728

Values are numbers (percentages) for categorical variables and means ± standard deviations, as appropriate. *p*-values comparing inborn and outborn infants by using chi-square test or Fisher’s exact test for categorical variables and Student’s *t*-test or Wilcoxon rank sum test for continuous variables. AS, Apgar score; BT, body temperature.

**Table 2:** The short-term neonatal outcome.

Characteristics	Inborn (*n* = 2683)	Outborn (*n* = 312)	aOR (95% CI)	*p* Values
Total number of infants admitted to NICU	2683	312	-	-
Mortality before discharge	625/2683 (23.3%)	12/312 (3.8%)	0.073 (0.039, 0.138)	<0.001
RDS	2585/2683 (96.3%)	302/312 (96.8%)	1.098 (0.546, 2.209)	0.794
Sepsis	628/2676 (23.5%)	123/311 (39.5%)	1.891 (1.482, 2.412)	<0.001
PDA, requiring surgical treatment	433/2642 (16.4%)	102/312 (32.7%)	2.436 (1.872, 3.169)	<0.001
Severe brain injury	558/2523 (22.1%)	100/310 (32.3%)	1.510 (1.154, 1.977)	0.003
IVH grade 3 or 4	407/2523 (16.1%)	76/310 (24.5%)	1.470 (1.090, 1.982)	0.012
NEC ≥ 2	230/2660 (8.6%)	60/312 (19.2%)	2.351 (1.706, 3.239	<0.001
Moderate-to-severe BPD or death	1504/2683 (56.1%)	197/312 (63.1%)	1.162 (0.886, 1.523)	0.278
Severe ROP	527/2134 (24.7%)	126/298 (42.3%)	1.606 (1.199, 2.151)	0.001

Values are numbers (percentages) for categorical variables and means (standard deviation), as appropriate. *p*-values and aOR are calculated using binary logistic regression after adjusting gestational period and 5 min Apgar scores. Some infants do not perform tests before discharge (or death), so there is a difference in the total number of comparisons, indicating the denominator of each variable. NICU, neonatal intensive care unit; RDS, respiratory distress syndrome; PDA, patent ductus arteriosus; IVH, intraventricular hemorrhage; NEC, necrotising enterocolitis; BPD, bronchopulmonary dysplasia; ROP, retinopathy of prematurity, aOR, adjusted odds ratio; CI, confidence interval.

**Table 3:** The long-term neurodevelopmental outcome.

Characteristics	Inborn	Outborn	aOR (95% CI)	*p*-Values
Mortality after discharge up to follow-up *	11/2141 (0.5%)	13/222 (5.9%)	15.658 (6.840, 35.844)	<0.001
Combined mortality *	636/2141 (29.7%)	25/222 (11.3%)	0.283 (0.172, 0.465)	<0.001
Death or NDI ^†^	873/1505 (58.0%)	76/132 (57.6%)	1.095 (0.739, 1.623)	0.650
Total number of infants with complete assessment at 18–24 months ^‡^	869	107		
NDI ^‡^	233/869 (26.8%)	51/107 (47.7%)	2.412 (1.585, 3.670)	<0.001
Cerebral palsy (GMFCS ≥ 3) ^‡^	28/869 (3.2%)	15/107 (14.0%)	4.460 (2.249, 8.845)	<0.001
Developmental impairment ^‡^	230/869 (26.5%)	48/107 (44.9%)	2.238 (1.469, 3.408)	<0.001
Visual impairment ^‡^	2/869 (0.2%)	-	0.712 (0.087, 5.806)	0.751
Hearing impairment ^‡^	12/869 (1.4%)	3/107 (2.8%)	2.157 (0.593, 7.850)	0.244

Values are numbers (percentages). *p*-Values and aORs are calculated using binary logistic regression after adjusting gestational period and 5 min Apgar scores. Combined mortality includes infants who died before discharge and after discharge. NDI was defined when least one of the following situations were diagnosed: cerebral palsy, developmental impairment, visual impairment or hearing impairment. * Infants with follow-up loss were excluded: 222 outborn and 2131 inborn infants. ^†^ Infants who had completed follow-up assessment or confirmed whether alive or dead were included for analyses: 132 outborn and 1505 inborn infants. ^‡^ Only for infants who had completed follow-up assessment: 107 outborn and 869 inborn infants. NDI, neurodevelopmental impairment; GMFCS, Gross Motor Function Classification System; aOR, adjusted odds ratio; CI, confidence interval.

**Table 4:** Baseline characteristics of infants who were not evaluated at 18–24 months of corrected age.

Characteristics	Infants Included in Analysis (*n* = 1637)	Infants without Follow-Up Data (*n* = 1358)	*p*-Values
**Maternal Characteristics**			
Maternal age, years	33.0 ± 4.2	32.6 ± 4.3	
Maternal age > 35 y	389 (23.8%)	314 (23.1%)	0.680
Primipara	994 (60.7%)	731 (53.8%)	0.000
Assisted conception	401 (24.5%)	265 (19.5%)	0.001
Multiple pregnancy	546 (33.4%)	410 (30.2%)	0.065
Maternal Hypertension	207 (12.6%)	144 (10.6%)	0.084
Maternal diabetes	104 (6.4%)	99 (7.3%)	0.310
Oligohydramnios	235 (16.0%)	140 (11.5%)	0.001
Any antenatal steroid	1275/1605 (79.4%)	1063/1336 (79.6%)	0.933
Chorioamnionitis	610 (44.3%)	493 (43.9%)	0.870
Premature rupture of membrane	332 (20.9%)	273 (20.7%)	0.928
Delivered by Caesarian section	1179 (72.0%)	942 (69.4%)	0.112
**Infant Characteristics**			
Birth weight, gram	850.3 ± 244.5	970.5 ± 224.0	<0.001
Small for gestational age	164 (10.0%)	70 (5.2%)	<0.001
Gestational period, week	26^0/7^ ± 1^5/7^	26^6/7^ ± 1^3/7^	<0.001
Male gender	843 (51.5%)	716 (52.7%)	0.503
Outborn	132 (8.1%)	180 (13.3%)	<0.001
AS, 1 min	3.5 ± 1.8	4.1 ± 1.8	<0.001
AS, 5 min	5.8 ± 2.0	6.4 ± 1.7	<0.001
AS < 7 at 5 min	916/1628 (56.3%)	582/1345 (43.3%)	<0.001
BT at admission	36.0 ± 0.8	36.1 ± 0.6	<0.001
pH at admission	7.25 ± 0.13	7.27 ± 0.11	<0.001

Values are numbers (percentages) for categorical variables and means (standard deviation), as appropriate. *p*-values comparing inborn and outborn infants by using chi-square test or Fisher’s exact test for categorical variables and Student’s *t*-test or Wilcoxon rank sum test for continuous variables. AS, Apgar score; BT, body temperature.

## Data Availability

The data that support the findings of this study are available from KNN but restrictions apply to the availability of these data, which were used under license for the current study, and so are not publicly available.
